# Sawdust and Bark-Based Substrates for Soilless Strawberry Production: Irrigation and Electrical Conductivity Management

**DOI:** 10.1371/journal.pone.0154104

**Published:** 2016-04-21

**Authors:** Claire Depardieu, Valérie Prémont, Carole Boily, Jean Caron

**Affiliations:** Département des sols et de génie agroalimentaire, Université Laval, 2480 boulevard Hochelaga, Québec, G1V 0A6 Canada; Institute for Sustainable Plant Protection, C.N.R., ITALY

## Abstract

The objective of this work was to optimize a soilless growing system for producing bare-root strawberry transplants in three organic substrates. Three trials were conducted in the Quebec City area to determine the productivity potential of a peat-sawdust mixture (PS25) and an aged bark (AB) material compared to conventional coconut fiber (CF) substrate. A first experiment was carried out to define appropriate irrigation set points for each substrate that allowed optimal plant growth and fruit yields. For all substrates, wetter conditions (irrigation started at -1.0 kPa for CF; -1.5 kPa for AB and PS25, relative to -1.5 kPa for CF; -2.5 kPa for AB and PS25) enhanced plant growth and fruit production. The second trial was carried out to test the productivity potential for commercial production of the three substrates using high-tunnels. After the addition of an initial fertilizer application to PS25, we successfully established bare-root plants that gave similar fruit yields than those in CF and AB. The productivity potential of PS25 and AB were further confirmed during a third trial under greenhouse conditions. The critical factor for plant establishment in PS25 was attributed to consistent N, P and S immobilization by microorganisms, as well as the retention of other elements (Mg^2+^, K^+^) in the growth media. Taken together, our results showed that PS25 and AB are promising alternative substrates to coconut coir dust for strawberry cultivation. This paper also provides a useful guide for strawberry cultivation in Quebec, and suggests future research that might be conducted to optimize soilless systems for cold-climate strawberry production in Northern America.

## Introduction

Strawberry (*Fragaria* × *ananassa*) is one of the most widely consumed fruit in the world, with annual sales that reached 1,533,000 tonnes in 2013 [1]. North American production of strawberries represents more than 25 percent of world production, with over 1,300,000 tons of fruit being produced every year [2]. Strawberries are mainly grown in California, Florida and Quebec with the latter being the largest area of strawberry production in Canada [3]. While the winter climate in Northeastern America is too cold for strawberries, its summer climate is appropriate for crop culture and provides an opportunity for local production. The development of protected cultivation systems allows offseason production of strawberry crops [4,5]. This is because strawberry production in soils is still facing challenges due to soil pathogens, herbicide injury and high labour costs [6–7]. Accordingly, strawberry production using soilless substrates represents a good alternative to field production of strawberries, and offers many advantages to growers by eliminating the need for chemical fumigations, crop rotations and non-fumigant soil disinfestations [8]. First introduced in the mid-80s, soilless growing systems for strawberries are now widespread in Europe but still remains in their early stages in North America [9].

Due to increasing risks of water scarcity resulting from climate change and the intensification of crop production worldwide, the agricultural sector needs to improve water use efficiency for fruit crops [10]. At present, soilless cultivation under protective conditions is an intense cultivation method that can provide more efficient use of water and fertilizers [11]. In addition, water management through precision irrigation and fertigation is of increased interest for the commercial production of field crops that respond positively to control water stress at critical growth stages [12]. The development of precision irrigation technologies provides opportunities to improve water use efficiency, and these allow the opportunity to provide exact amounts of water at the right times to meet crop water requirements [13]. This new approach to irrigation management is currently under development for intensive field production of strawberries in specific areas of Europe [14]. Similarly, fertigation using drip-irrigation systems allows a systematic approach to effective water management and timely fertilizer application [15]. Strawberries have high water requirements and are salt sensitive crops, so the control of both water use efficiency and irrigation water quality is critical for them [16]. Irrigation water quality is usually monitored by measuring electrical conductivity (EC) [17]. Previous studies have reported that an EC in excess of 2.5 dS m^-1^ had a negative impact on plant growth, fruit quality and yield for strawberries grown in soil [18]. Opposingly, higher fruit yields, better quality and higher strawberry weights were obtained at EC 2.5 dS m^-1^ than those obtained at lower EC values when using Trico irrigation under soilless conditions [19].

The increasing demand for low cost, environmental friendly and highly performing soilless substrates for crop production has led to the search for alternative materials as constituents of growth media such as organic wastes from the agri-food and agriculture industries. For the hydroponic production of strawberries, peat-based and rockwool growing media are popular in Europe, while untreated sawdust, coconut fiber, bark and perlite are widely used in North America [20]. Organic substrates are usually preferred because of their low costs, biodegradability and their high productivity potentials [21,22]. In Canada, mill residues such as sawdust and bark are the cheapest source of biomass [23] and sawdust has been widely used in commercial plant production for decades [24]. Despite good air contents and high saturated hydraulic conductivities, these wood industry by-products have low water retention capacities. Several studies reported limitations to plant growth and fruit production with these growing media that was attributed to low water availability [25], insufficient aeration of growth media caused by microbial activity, or inappropriate particle-size distribution [26,27], and nutrient immobilization [28], as well as negative effects due to salt and toxic compound accumulations [29]. Even if used mixed with peat, sawdust can still have a negative impact on strawberry fruit production compared to those for the use of peat and peat-bark substrates [30]. However, a recent study conducted in soiless tomato production in Quebec revealed that a local peat-sawdust mix is a promising replacement for rockwool provided appropriate and constant irrigation trigger sets were defined [31]. To date, the productivity potential of this substrate has not been tested for the greenhouse production of strawberries.

The purpose of this research was to evaluate three selected organic substrates for the greenhouse production of strawberries in Quebec. It was of particular interest to characterize one locally produced, low-cost peat-sawdust substrate with respect to its physical, chemical properties and productivity potentials using bare-root plants. Specifically, the objectives for this study were to: (1) evaluate the effects of water potential set points and substrate type on bare-root strawberry plant establishment, early growth and yields during a short-term experiment (Experiment 1); (2) compare the productivity potential of the three substrates within a commercial greenhouse facility (Experiment 2); and (3) compare two different fertigation strategies (Experiment 3). The following questions were asked: (1) Could a peat-sawdust substrate have similar productivity potential to that of coconut fiber or bark substrate? and (2) Would doubling the fertilizer level in the irrigation solution relative to the recommended EC of 0.6–1.8 dS m^-1^ throughout the crop production [32] improve fruit quality and yields?

## Methodology

### Growing media composition and preparation

A peat-sawdust substrate (PS25), a coir fibre (CF) and an aged bark (AB) growing media were tested in this study. The peat-sawdust substrate was a mixture of 30% of white spruce sawdust [*Picea glauca* (Moench) Voss.] sieved to morethan 6 mm, and 70% of brown sphagnum peat (Experiments 1 and 2: BRO MOSS®; Fafard et Frères ltée., Saint-Bonaventure, QC, Canada—Experiment 3: H4-H5 von Post scale; Premier Tech, Riviere-du-Loup, QC, Canada) sieved to less than 0.5 mm. In the manufacturing sequence, the substrates were pH adjusted to 5.5 and saturated with a full nutrient solution. The commercial substrate AB was a mixture of aged bark, sphagnum peat moss, sand and compost which was mainly containing animal manure and a small fraction of plant material (AGRO MIX® N7, Fafard et Frères ltée., Saint-Bonaventure, QC, Canada) and received an initial fertilizer load at the manufacturing facility. The commercial CF growing media was made of a natural fibre extracted from the husk of coconut (Groupe Horticole Ledoux Inc., Sainte-Hélène-de-Bagot, QC, Canada). This material came in grow bags (Experiments 1 and 2: Performa Globalys aerated; 72 cm. 19 cm. 13 cm—Experiment 3: Ultima Millenium; 100 cm. 18 cm. 13 cm) that were directly used for root cuttings. Before planting, dry slabs of coconut fiber were saturated with a solution of calcium nitrate (2.5–3 dS m^-1^) according to the manufacturer’s instructions and the pH of the solution adjusted to around 5.8. After saturation, all substrates were drained for 24 h to facilitate water retention. Due to its good capillary rise, the PS25 substrate was laid on a capillary mat (AQUAMAT^®^, Soleno Textiles, Laval, QC, Canada) to reduce the amount of applied nutrient solution [33].

### Physical characteristics of growing media

#### Particle-size distribution

Particle-size distribution (PSD) was obtained by hand sieving particles of dried substrates (sieve openings: 16, 8, 4, 2, 1, 0.5, 0.25 and 0.1 mm) for three minutes and weighing the material retained by each sieve. The equivalent pore-size distribution was expressed in function to the substrate matric water potential (*h*) according to Jurin’s law:

$$d_{eq}=-\frac{h}{4\upsilon \;\text{cos}\;\alpha }$$

where *d*_*eq*_ is the equivalent size of the pore, *υ* is the surface tension of water (75.10^−3^ Nm^-1^) and α is the contact angle of water with the pore walls, assumed to be 0.

#### Saturated hydraulic conductivity, water retention curves and bulk density

The saturated hydraulic conductivity (K_sat_) was determined using the vertical constant head method utilizing a Mariotte bottle [34]. Blocks were cut out from growth media and compacted into flow cells at the same bulk density used for the generation of the water retention curves (WRC). Samples were brought to saturation and maintained fully saturated for 24–48 h. The water flux was measured under steady state conditions and K_sat_ was calculated using the Darcy's Law.

WRC were generated using a method developed by the Conseil des productions végétales du Québec [34] with the following modifications. Water content at saturation was measured with a domain reflectrometry (TDR) probe inserted vertically into the substrate. Volumetric water content (q) was further calculated from the dielectric constant value (*Ka*) obtained by TDR readings using an equation developed by Caron and collaborators [35], for *Ka* values ranging 49–64:

q=0.0042*Ka+0.6635

Subsequently, samples were allowed to drain for 2 h, weighed and placed on a tension table. The matric potential was adjusted using the tension tables and measured using a tensiometer inserted vertically at the middle-height of the sample. Water retention curves were generated by using the weight-loss method (desorption curves) and the weight-gain method (sorption curves) as previously described [34].

Water retention curves obtained from the experiment were fitted using the van Genuchten model [36]:

$$\theta =\theta_{r}+(\theta_{s}-\theta_{r}){\left(1+\alpha {\left|h\right|}^{\text{n}}\right)}^{-\text{m}}$$

where *h* is the matric potential (cm), *θ*_*r*_ is the residual water content (cm^3^. cm^−3^), *θ*_*s*_ is the water content at saturation (cm^3^. cm^−3^) and *α*, *n*, and *m* are empirical parameters. The air volume content or air-filled porosity (AFP; corresponding to water losses between 0 and -1 kPa), the water holding capacity (WHC; defined for the range of *h* from -1 to -10 kPa) and the water buffering capacity (WBC; water losses between -5 and -10 kPa) were then deduced from the desorption curves.

Samples used to determine water retention characteristics were further oven-dried at 105°C for 24 h and weighted. The bulk density was determined according to the core method [37].

### Chemical characteristics of substrates

During cultivation, substrate solution was extracted at mid-height of a container using a suction lysimeter (Model Soil water sampler 1905, Soil Moisture Equipment Corp., Santa Barbara, CA, USA). Sampling was carried out in triplicate for each treatment. The pH_lys_ and EC_lys_ of the collected solutions were then measured using a pH meter (Symphony SB70C; VWR, Mont-Royal, Quebec, Canada) and a conductivity meter (Symphony, 11388–382 Epoxy: VWR, Mont-Royal, QC, Canada). At the end of the experiment, the growing media was divided into three equal parts along the container depths. The pH_SSE_ and EC_SSE_ were measured on Substrate Saturated Extracts [38]. The pH_SSE_ and pH_lys_ values obtained for the three experiments were within the acceptable ranges for the plant growth of strawberries.

Cation and anion analyses were carried out for the substrate solution extracts obtained using a suction lysimeter. K, Ca, Na and Mg contents were determined with a flame atomic absorption system (Model 3300, Perkin Elmer, Uberlingen, Germany). Cl^-^ and NO_3_-N contents were determined using a high-performance liquid chromatography system with conductivity detection (Waters 340, Millipore Corporation, Milford, MA, USA). NH_4_-N (Berthelot Reaction) and P (Murphy and Riley) contents were determined by colorimetry using a conventional optical spectrophotometer (HITACHI U-1100). Chemical analyses were carried out in triplicates.

### Experimental design and plant growth conditions

Experiment 1 (Exp. 1) was conducted from April 11 to June 21, 2011 in the high performance EVS greenhouses at Université Laval, Quebec City, Canada (lat. 46°77’56” N, long. 71°28’29” W). The experimental design was a completely randomized block design composed of six blocks and a total of 48 experimental units (EU). Each EU was made up one plastic bag or 3 containers at a density of nine plants. Bare root *Fragaria × ananassa* Duch. Seascape plants were transplanted into coco fibre grow bags (18 L; 72 cm. 19 cm. 13 cm; Performa Globalys; Groupe Horticole Ledoux, Sainte-Hélène-de-Bagot, QC, Canada) whereas three plants were inserted into plastic containers (10.7 L, 27.3 cm. 22.2 cm; ITML model NCS03000, Myers Industries Lawn & Garden Group, Middlefield, OH, USA) containing peat-sawdust substrates or aged bark substrates. The planting density was of 24.5 plants. m^-2^. In the greenhouse, a PRIVA system (Priva B.V., Vineland Station, ON, Canada) controlled the climate with a day/night temperature of 21–23/17°C and a relative humidity of 50%. Daily, artificial lighting provided by high vapour pressure sodium lamps (600 W; PL Light Systems Canada Inc., ON, Canada) was automatically switched on at 10:30 a.m. and switched off at 4 p.m. However, supplemental lighting was turned off when the photosynthetically active radiation (PAR) exceeded 1200 umol m^-2^ s^-1^. Plants were micro-irrigated using a drip irrigation system (Netafim USA Ltd., Fresno, CA, USA). Each plant was equipped with an angle barber stake connected to a dripper (one dripper per plant; 2 L. h^-1^ WPJC) and irrigated with a nutrient solution adapted to plant development.

Experiment 2 was carried out in a multi-tunnel greenhouse (112 m x 8 m) located at a commercial strawberry farm (46°90’13” N,70°93’70”W; Saint-Jean-de-l’Île-d’Orléans, QC, Canada), from May 30 to October 12, 2011. Plants were grown in containers (8 L; model HB1200C, Concept Plastics Limited, Mississauga, ON, Canada) and growing bags (50 cm. 18 cm. 13 cm) that were supported by north-south oriented wooden structures at 1 m above ground. The experimental design consisted of 3 blocks and 12 EU. Each EU was composed of 41 grow bags (CF) or 123 containers (PS25 and AB) with a total of 369 plants. The planting density was 10 plants m^-1^. Plants were micro-irrigated using a pressure compensated drip system (PCAS), with one dribble ring per container (Model #PCR85-24; Dramm Corporation, Manitowoc, WI, USA) or three angle barber stakes connected to drippers (4 L h^-1^ WPJC, Netafim USA Ltd., Fresno, CA, USA).

Experiment 3 was carried out in the high performance EVS greenhouses at Université Laval, Quebec City, Canada (lat. 46°77’56” N, long. 71°28’29” W) from January 27 to April 30, 2012. The experimental design consisted of six blocks and 36 EU. Each EU was made up one grow bag and three naked-root seedlings were planted in CF (11.7 L, 50 cm. 18 cm. 13 cm) or in PS25 and AB medium (15 L, 41 cm. 20 cm. 18 cm). Growing bags were placed into north-south oriented slabs at 75 cm above ground for a planting density of 9.4 plants m^-2^. In the greenhouse, day/night temperature and relative humidity were maintained at: (1) 16/12°C and 60% from January 27 to March 23, 2012 and (2) 19–20/12–14°C and 50% from March 24 to April 30, 2012. Artificial lighting was provided by high vapour pressure sodium lamps from 9:00 a.m. to 9:00 p.m. The irrigation solution was distributed using a drip-tape system (Chapin Twin-Wall Deluxe 5/8”, Jain Irrigation Systems Ltd.; Jain Irrigation Inc., Fresno, CA, USA). Growing bags were individually irrigated by six emitters distributed on two lines of drip-tape, with a discharge rate of 0.74 L h^-1^ per emitter.

### Fertigation coupled with substrate moisture-based irrigation

Irrigation scheduling was based on substrate matric water potential measured using wireless tensiometers (Hortau model TX3, Levis, Canada) inserted vertically in the rooting zone at mid-substrate height. For each treatment, three tensiometers were installed into three independent blocks. Media matric potential was measured regularly and irrigation was triggered once the matric potential threshold was reached. During the Exp.1, two irrigation set points were tested for each substrate corresponding to two treatments; one relatively wet (I1) and one relatively dry (I2). Irrigation was initiated when substrate matric water potential reached: -1.5 kPa and -2.5 kPa for PS25 and AB; -1.0 kPa and -1.5 kPa for CF. Irrigation set points for Exp. 2 were determined based on the preliminary results from Exp. 1 and adjusted with plant development: -1.0 kPa for CF and -1.5 kPa for PS25 and AB during the establishment period; -1.0 kPa for CF and -1.8 kPa for PS25 and AB during fruit production. In Exp. 3, the matric potential thresholds were -1.2 kPa for CF and -1.5 kPa for PS25 and AB.

Fertigation was conducted according to CTIFL instructions [39] (S1 Table). The EC of the irrigation solution was initially set to 0.6 dS m^-1^ and progressively increased to 1.6 dS m^-1^ during the establishment period, then further maintained at 1.2 dS m^-1^ during the flowering and fruit production period. In Exp. 3, two fertilizer treatments were carried out; one with the recommended fertilizer rate by CTIFL (F1) and one with a fertilizer rate (F2) at a concentration twice that of F1. During each experiment, daily control of the EC and pH of the applied nutritive solution and leachates was performed across the EU in three blocks. Irrigation and leaching volumes were collected to check its composition and to make sure that drainage volume represented about 20% of applied volume during cultivation [16,32]. The duration of irrigation was adjusted to avoid salt accumulation in the leachate. For all treatments, the pH of the nutrient solution ranged 5.0–6.6.

### Fruit production, crop yield and fruit quality

The number of fruit was determined every week during the plant establishment period. Every 3–4 days, fresh fruit was harvested at its proper maturity stage, classified into marketable or unmarketable groups, and weighted. Final dry masses of leaves, stems and roots were determined after drying in a thermo-ventilated oven at 65°C until constant mass was reached. In experiments 2 and 3, fruit quality parameters including caliber, firmness (penetrometers FT02, QA Supplies LLC) and total fructose level (Brix index; refractrometer PAL-1, Atago) were measured weekly. The average fruit size was calculated as the ratio of the marketable fruit weight by the fruit number.

### Statistical analysis

Data were analysed with the MIXED procedure of SAS 9.3 (SAS Institute, Inc., Cary, NC). Data normality was tested using the Shapiro-Wilk test (*P*>0.01). If necessary, non-normally distributed data were transformed to normality. Homogeneity of variance was analyzed by visual inspection of residuals plot. The least square means were compared when the ANOVA model was significant at *P* = 0.05.

## Results and Discussion

### Physical characterization of growing media

#### Initial characteristics

**Fig 1:**
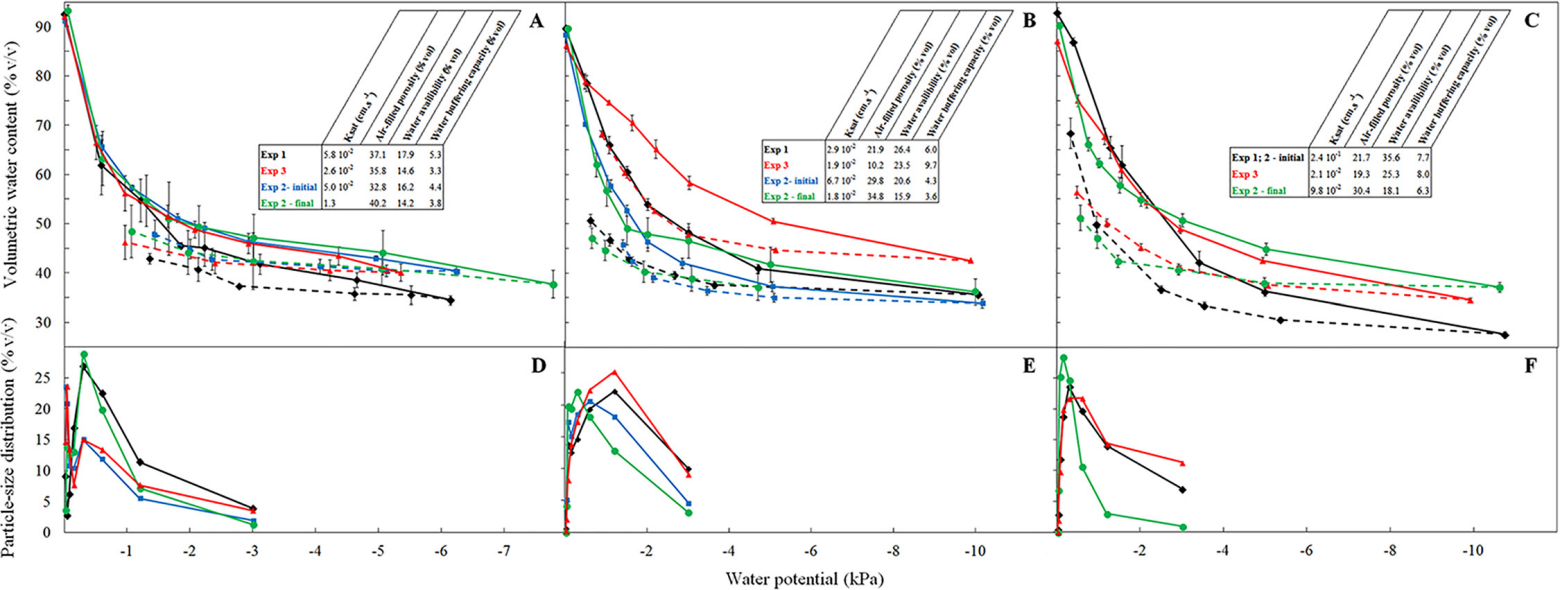
Water retention curves (**A, B, C**) and calculated water potential corresponding to size distribution (**D, E, F**) of CF (**A, D**), AB (**B, E**) and PS25 (**C, F**) substrates. Each point is the mean (n = 3) with standard deviation (SD). For each WP value (kPa), the corresponding particle size (mm) are as follow: WP = -3, [0,25; 0,1[; WP = -1.2, [0.5; 0.25[; WP = -0.6, [1.0; 0.5[; WP = -0.3, [2.0;1.0[; WP = -0.15, [4.0; 2.0[; WP = -0.08, [8.0; 4.0[; WP = -0.04, [16.0; 8.0[; WP = -0.02, >16.0.

The three substrates used in this study differed significantly in their initial hydraulic properties (Fig 1). Their water retention curves (WRC) revealed that CF is a highly porous growing media with a high air-filled porosity (32.8–37.1% v/v) with acceptable water availability (17.8–23.1% v/v), in agreement with previous data obtained for coconut growing media [40, 41]. Due to a higher proportion of fine particles than CF, AB was less aerated than CF (Fig 1, Table 1) and exhibited a higher water holding capacity (ranging 24.9–32.3% v/v) in combination with a low K_sat_ values (0.02–0.07 cm s^-1^). Initial physical properties of AB indicated that this substrate presents values of air filled porosity above the lower limit (air-filled porosity above around 20% v/v) but presented a potential risk of hypoxia to the root system in one case only (Exp. 3, see Table 1) that can result in plant growth limitations for that specific experiment [26]. The peat/sawdust substrate PS25 was a porous growing media (air-filled porosity above 20% v/v) with high water availability (33.3–43.2% v/v), consistent with previous observations made for this mix [31].

**Table 1:** Initial and final substrate physical properties of CF, AB and PS25 substrates. The air-filled porosity, the water availability and water buffering capacity were calculated from the WRC. In the experiment 3, means for each substrate are reported since fertilization levels had no significant effects on the final K_sat_ and bulk density. Different letters indicate significant differences at P = 0.05. -: variable not measured.

		Saturated hydraulic conductivity (Ksat, cm.s-1)		Bulk density(BD, g.cm-3)		Air-filled porosity (AFP, % vol)		Water availability (WHC, % vol)		Water buffering capacity (WBC, % vol)	
Experiment	Substrate	Initial	Final	Initial	Final	Initial	Final	Initial	Final	Initial	Final
**Exp. 1**											
** **	CF	0.057 (0.021) b	-	0.095 (0.007) b	-	37.07 (3.87) a	-	23.11 (2.50) c	-	5.25 (1.13) b	-
	PS25	0.236 (0.032) a	-	0.084 (0.002) c	-	21.66 (3.55) b	-	43.25 (1.89) a	-	6.01 (0.31) ab	-
** **	AB	0.029 (0.005) b	-	0.236 (0.002) a	-	21.87 (1.97) b	-	32.37 (0.55) b	-	7.69 (1.11) a	-
	***P value***	< .0001		< .0001		0.002		0.001		0.047	
** **	***Contrasts***										
	CF-PS25	0.001		0.029		0.001		0.002		0.019	
** **	CF-AB	0.401		< .0001		0.001		0.024		0.358	
**Exp. 2**											
** **	CF	0.05 (0.04) d	1.29 (0.87) a	0.100 (0.007) b	0.099 (0.012) b	32.81 (0.37) b	40.22 (7.04) a	20.58 (1.07) c	17.94 (1.73) d	4.43 (1.05) b	3.76 (0.76) b
	PS25	0.24 (0.06) b	0.10 (0.05) bcd	0.081 (0.002) c	0.084 (0.003) c	21.66 (3.55) c	34.44 (2.33) b	43.25 (3.27) a	24.43 (0.38) b	6.01 (0.31) a	6.33 (0.13) a
** **	AB	0.07 (0.01) cd	0.18 (0.01) bc	0.203 (0.003) a	0.236 (0.002) a	29.76 (1.33) b	34.75 (1.54) ab	24.91 (0.96) b	19.45 (0.72) cd	4.28 (0.37) b	3.57 (0.91) b
	***P value***										
** **	Substrate (S)	0.417		< .0001		0.001		< .0001		< .0001	
	Date (D)	0.004		0.399		0.001		< .0001		0.034	
** **	S*D	0.001		0.549		0.642		0.4844		0.718	
	***Contrasts***										
** **	CF-PS25	0.505		0.027		0.003		0.001		< .0001	
	CF-AB	0.192		< .0001		0.163		0.443		0.74	
**Exp. 3**											
	CF	0.263 (0.101) a	1.326 (0.493) a	0.102 (0.005) d	0.112 (0.015) d	35.77 (3.37) a	-	17.83 (1.27) b	-	3.26 (0.64) c	-
** **	PS25	0.021 (0.0002) c	0.057 (0.028) b	0.168 (0.007) c	0.223 (0.05) b	19.29 (1.71) b	-	33.29 (0.83) a	-	8.02 (0.33) b	-
	AB	0.019 c	0.038 (0.010) b	0.349 (0.03) a	0.326 (0.038) a	10.24 (1.29) c	-	33.17 (0.43) a	-	9.71 (0.92) a	-
** **	***P value***										-
	Substrate (S)	< .0001		< .0001		< .0001		0.0002	-	< .0001	
** **	Date (D)	< .0001		0.135		-	-	-	-	-	-
	S*D	0.003		0.057		-	-	-	-	-	-
** **	***Contrasts***										
	CF-PS25	< .0001		< .0001		0.0001		0.001		0.0001	
** **	CF-AB	< .0001		< .0001		< .0001		0.0003		< .0001	

#### Time evolution of physical properties

After long-term cultivation of strawberries, a significant decrease in water availability was observed for all substrates (Table 1), which was associated with a marked increase of the air-filled porosity in PS25 and CF growing media. These results are consistent with previous data obtained using peat/sawdust mixtures [27] and coconut coir dust [41]. The higher proportion of large particles after plant growth on substrates (Fig 1) may be due to a loss of fine material for irrigation or increased root activity modifying the substrate.

Taken together, the initial and final characteristics did not suggest any effect of aeration properties (but in Exp. 3, see Table 1) on substrate performance. In addition, irrigation frequency was adjusted to maintain the air-filled porosity within the optimal range for each substrate, therefore integrating any differences that substrate physical properties, container height and volume may have had on crop performances and investigation of substrate differences hence focused on other aspects.

### Experiment 1

#### Chemical properties of the growing media during plant establishment

The EC_lys_ was significantly different for each substrate; CF had the highest EC while PS25 had the lowest (Table 2). Through the cropping season, the EC_lys_ was in an acceptable range for plant growth [32]. The EC_SSE_ measured at three different depths revealed a salt accumulation in the upper layer of the growing media for PS25 and AB, in contrast with the CF substrate which was packed in bags (Table 2). Salt build-up at the surface is generally caused by the surface evaporation, the irrigation system used, and/or the presence of an immobile water phase [42]. Given the high proportion of immobile water phase observed in peat-sawdust media [42], we expected this result for PS25. However, the salt accumulations found with AB may indicate significant surface evaporation. Starting from these results, white plastic films were attached to the top of the containers to reduce surface evaporation in Exp. 2 and 3.

**Table 2:** Chemical properties of the three substrates. The electrical conductivity was measured on substrate solution extracted using a suction lysimeter (EC_lys_) or the SSE method (EC_SSE_) at three different substrate depths (T: top; M: medium; B: bottom). No significant effect of time was obtained for EC_lys_. Different letters indicate significant differences at P = 0.05.

Substrates	EC_lys_ (dS m^-1^)	EC_SSE_ (dS m^-1^)		
Time	0–72	72		
Depth	-	T	M	B
**CF**	1.41 (0.49) a	0.86 (0.14) b	0.78 (0.12) b	0.71 (0.08) b
**PS25**	0.53 (0.17) c	0.46 (0.12) c	0.26 (0.05) d	0.25 (0.02) d
**AB**	0.97 (0.18) b	1.30 (0.30) a	0.74 (0.08) b	0.71(0.13) b
***P value***				
Substrate (S)	< .0001	< .0001		
Irrigation (I)	0.018	0.451		
S*I	0.005	0.339		
Depth (D)	-	< .0001		
S*D	-	0.001		
***Contrasts***				
CF-PS25	< .0001	< .0001		
CF-AB	< .0001	0.010		

#### Effects of irrigation regimes and substrate type on short-term performance of PS25, AB and CF substrates for plant growth and fruit production

**Fig 2:**
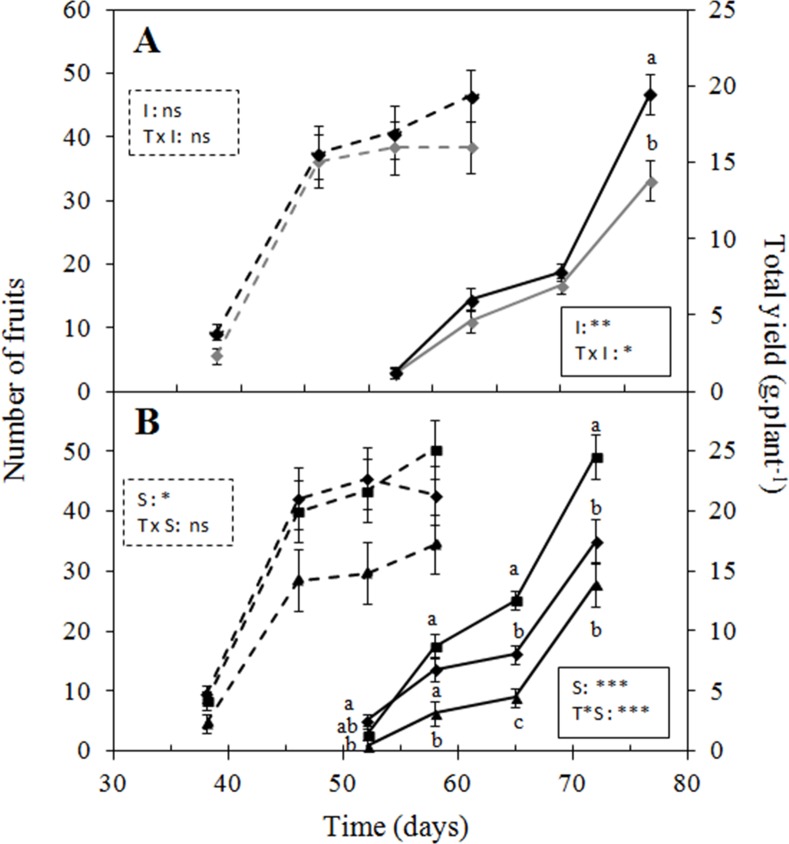
Evolution of fruit production (dotted lines) and total yield (plain lines) of strawberry plants grown under two irrigation treatments (**A**) and in different substrates (**B**). (**A**) Variation in the two parameters under relatively wet conditions (black lines) and relatively dry conditions (grey lines). (**B**) Number of fruits and total yield obtained from plants grown in CF (diamonds), AB (squares) and PS25 (triangles). The P-values obtained from the generalized linear mixed model (GLMM) used to fit the data are reported for the irrigation threshold (I), substrate (S) and time (T) effects, as follows: ns: no significant; (*) = P<0.05; (**) = P<0.01; (***) = P<0.001. Significant levels of the post hoc test results are indicated by letters. Each point represents the mean (n = 3) with SD.

The temporal evolution of fruit production and total yield is presented in Fig 2. Total yields were compared between AB and PS25, with CF as the reference substrate. For all substrates, irrigation regimes had no effect on berry production at the early stage of plant establishment (0–58 d); however, total yield was significantly affected by irrigation treatments by the end of the experiment (day 72; Fig 2A). These results indicate that the maintenance of wetter conditions was beneficial for plant establishment and possibly for the production of larger fruits, and in line with previous studies [43, 44]. The relatively dry treatment resulted into lower leaf dry mass only for plants grown in PS25 (Table 3), suggesting that water availability may be more critical for plant growth in PS25 than in CF and AB. At the early stages of plant establishment, fruit production and total yield were significantly lower for plants grown in PS25 (Fig 2B). The higher yields obtained for AB compared with those for CF may be explained by the production of larger fruits, since an equivalent fruit production was observed for the two substrates. Both cumulative marketable yield and final leaf dry mass were higher for plants grown in AB, followed by those grown in CF and PS25 (Table 3). The lower EC_lys_ values obtained for PS25 (Table 2) suggest a decreased nutrient availability that might have caused plant growth limitations. Phytotoxic effects with sawdust substrates as a result of nutrient immobilization or microbial competition for nutrients has been previously reported to impact plant growth at initial crop developmental stages [26, 45]. The temporal pattern of both fruit production and total yield for PS25 suggests that nutrient immobilization occurred at the early stage of bare-root plant establishment (Fig 2B). Though composts generally contain enough nutrients to negate the initial use of supplemental fertilization, our results highlight the need for an initial fertilizer application to PS25 for successful plant establishment. Based on these observations, PS25 received an initial fertilizer load in Exp. 2 to fulfill initial nutrient requirements of bare-root plants up to the formation of the first set of leaves (S1 Table).

**Table 3:** Cumulative unmarketable and marketable yields and leaf dry mass at the end of the experiment for strawberry plants grown in CF, AB and PS25 substrates. Significant differences between group means were determined by ANOVA at P = 0.05.

Substrates	Cumulative unmarketable yield (g. plant^-1^)	Cumulative marketable yield (g. plant^-1^)	Leaf dry mass (g.plant^-1^)	
Irrigation level	I1 and I2	I1 and I2	I1	I2
**CF**	6.25 (1.72)	28.62 (9.81) b	15.40 (2.63) b	15.56 (0.63) b
**PS25**	5.43 (1.68)	16.57 (8.15) c	12.48 (2.57) c	9.46 (1.32) d
**AB**	6.97 (2.92)	40.19 (9.47) a	18.18 (2.65) a	19.53 (1.40) a
***P value***				
Substrate (S)	0.335	<0.0001	< .0001	
Irrigation (I)	0.293	0.057	0.438	
S*I	0.066	0.178	0.027	
***Contrasts***				
CF-PS25	0.429	0.002	<0.0001	
CF-AB	0.485	0.002	<0.0001	

### Experiment 2

#### Chemical characteristics of the substrates

**Fig 3:**
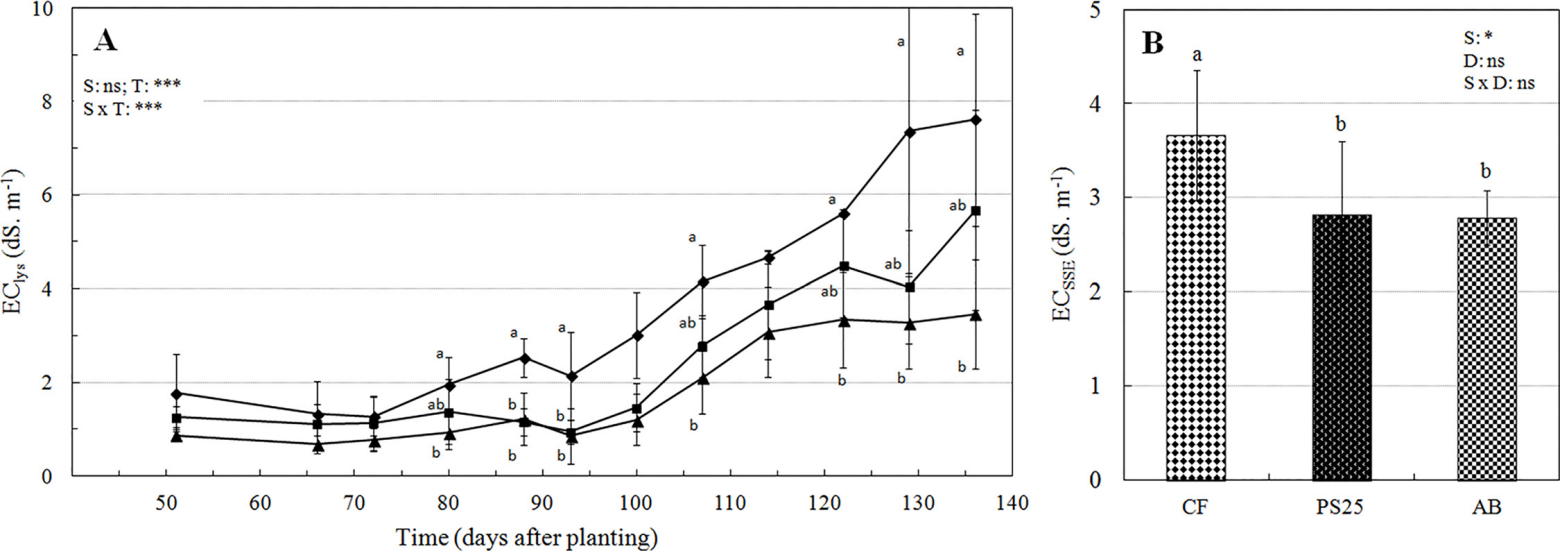
Chemical characteristics of the substrates. **(A)** Evolution of EC_lys_ in CF (diamonds), AB (squares) and PS25 (triangles) during the growing period. **(B)** EC_SSE_ measured at the end of the experiment. The P-values are reported for time (T), depth (D) effects and their interaction with substrate (S) as follows: ns: no significant; (*) = P<0.05; (**) = P<0.01; (***) = P<0.001. Significant differences between substrates are indicated by letters.

Chemical properties, including EC_SSE_ and EC_lys_, differed between substrates (Fig 3). Despite appropriate salinity management, the temporal variation of EC_lys_ revealed a progressive salt accumulation after 107 days (Fig 3A). Compared to PS25, the increase in EC_lys_ observed with CF may indicate the accumulation of salts, probably due an insufficient leaching of this substrate by the end of the experiment. However, neither the accumulation of fertilizers nor the acidification of this growing media had an impact on strawberry yield (data not shown), since their values remained in the optimal range for strawberry production and plant growth [32]. At the end of the experiment, EC_SSE_ was higher in CF compared to that in the other substrates (Fig 3B). In light of the results obtained in Exp. 1, we suspected that initial nutrient immobilization had a negative impact on the early growth of strawberry plants. The initial fertilizer application at the beginning of this trial allowed the maintenance of similar EC_lys_ values between substrates through 72 days. However, significant lower EC_lys_ values for PS25 compared to that of CF were observed after 134 days. Taken together, these results indicate that an initial fertilizer application to this substrate was beneficial for plant establishment, but might not be sufficient after 12 weeks of crop production.

#### Long-term performance of PS25 compared to AB and CF

In this trial, plants grown in PS25 were successfully established and gave similar fruit yields and fruit size compared to those of plants grown in AB and CF (Table 4). The average fruit size ranged between 11.72–14.77 g. berry^-1^ in accordance with previous observations with ‘Seascape’ [46]. Total strawberry yield and mean fruit weight did not differ for plants grown in CF, PS25 and AB, as previously reported in the literature [47, 48]. Similar strawberry yields were attained using the PS25 substrate while requiring less water compared to those with AB and CF (S2 Table). Given that the total dry mass did not differ among substrates, this result can be attributed to the use of the capillary mat with PS25. We obtained a cumulative marketable yield of 289–343 g. plant^-1^ for 135 days of cultivation, and this was equivalent to the yields obtained in previous trials with fall planted ‘Seascape’ strawberries grown in soils in western North Carolina [46], and in organic substrates in Quebec (Martine Dorais, personal communication). The monthly total yield obtained during this study (83.2–84.5g. plant^-1^) falls in the range of high tunnel yields obtained for soil-grown ‘Seascape’ plants in Utah high tunnels [49].

**Table 4:** Effect of the three organic substrates on the cumulative total and marketable yields, fruit quality parameters and final leaf dry mass.

Substrate	Cumulative total yield (g. plant^-1^)^a^	Cumulative marketable yield (g. plant^-1^)^a^	Fruit caliber (g plant ^-1^)^b^	Firmness^b^	Sugar content^b^	Leaf dry mass (g plant^-1^)
**CF**	323.39 (21.32)	288.95 (25.38)	12.64 (0.14)	282.74 (9.77)	8.62 (0.18)	23.16 (4.42)
**PS25**	371.84 (47.31)	332.40 (45.79)	13.91 (1.05)	287.17 (7.59)	8.12 (0.11)	24.16 (5.79)
**AB**	377.47 (21.32)	342.88 (17.98)	13.41 (0.51)	300.62 (5.42)	8.29 (0.28)	22.91 (3.81)
***P* value**						
* *	0.205	0.207	0.302	0.101	0.103	0.945
***Contrasts***						
CF-PS25	0.150	0.171	0.170	0.048	0.049	0.809
AB-PS25	0.829	0.709	0.225	0.525	0.521	0.764

^a^Significant differences between group means were determined by ANOVA at P = 0.05.

^b^Parameters analysed by repeated-measured ANOVA (Fall 2011, 34 harvests: from July14^th^, 2011 to October 11^th^) at P = 0.05.

### Experiment 3

#### Long-term performance of CF, AB and PS25 for greenhouse-grown strawberries

**Fig 4:**
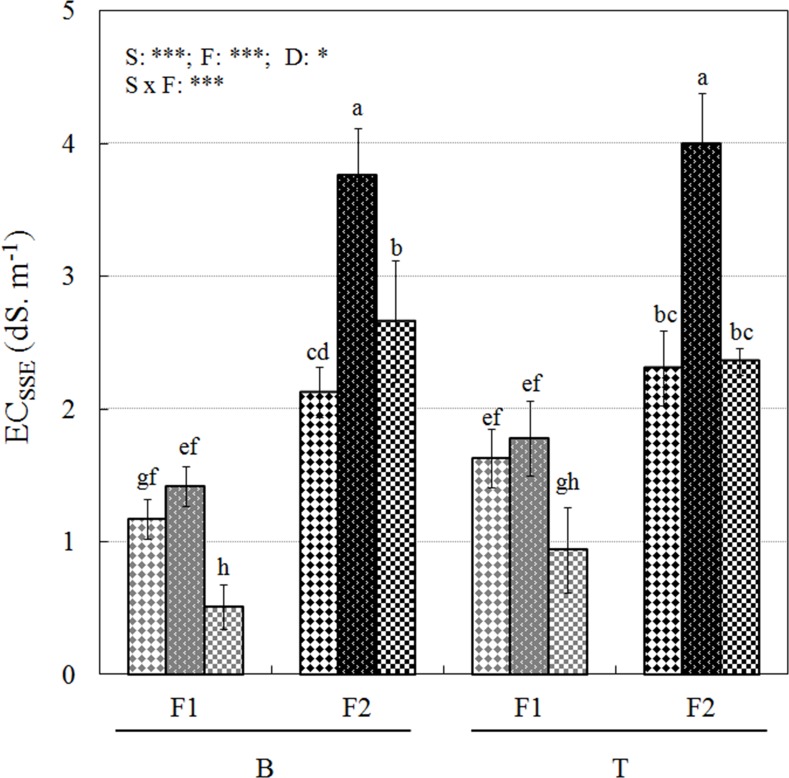
Final EC_SSE_ measured at two substrate depths (T: top; B: bottom), under two fertilization rates (F1, F2) for PS25 (diamonds), CF (circumflex) and AB (squares). Each point is the mean (n = 3) with SD. Significant P-values for fertilization (F), depth (D) and substrate (S) effects are reported as follows: (*) = P<0.05; (**) = P<0.01; (***) = P<0.001. Significant levels of the post hoc test results are indicated by letters.

Neither yields nor fruit quality were significantly affected by the fertilizer levels which ranged 2.9–6.1 mmol L^-1^ for F1 and 5.8–12.2 mmol L^-1^ for F2 during the experiment (Table 5). Similarly, fruit yield and firmness were not affected by an increase in nitrate concentration of the nutrient solution from 3.75 to 15 mmol L^-1^ [50, 51]. The mean berry weights of 10.70–13.85 g (Table 5) were similar to fruit caliber found with greenhouse-grown ‘Seascape in soilless culture [52]. The strawberry plants grown in the AB substrate exhibited an increased leaf dry mass and gave higher yields than those grown in PS25 and CF, suggesting that the higher bulk density and the lower air-filled porosity for that substrate did not affect yields or was compensated by another factor. It therefore appears that plant growth restriction in CF and PS25 may be explained by several other factors including nutrient immobilization and the CEC characteristics of the different substrates. In soilless culture, high CEC allows easy storing and releasing of individual nutrients. In our study, EC_SSE_ values showed a lower reserve of nutrients in CF and PS25 as compared to those in AB at the end of the experiment (Fig 4). While *Sphagum* peat-based substrates have high CECs, sawdust-based materials generally exhibit low CECs [53]. Given that PS25 is a mixture of 75% *Sphagum* peat moss and 25% sawdust, we expected this substrate to have an intermediate or a high CEC [41]. Finally, higher nutrient immobilization rates combined with lower nutrient reserves may explain the lower yields obtained for plants grown in PS25. For CF, the lower EC_SSE_ values combined with a high Na^+^ and Cl^-^ contents, despite leaching performed according to standard, may have had an effect though.

**Table 5:** Effect of substrate type on average total yield, marketable yield, fruit quality parameters and final leaf dry mass. (Fall 2012, 18 harvests: from March 23th, 2012 to May 2^nd^, 2012). Two fertilizer rates were tested, with the second fertilizer rate (F2) at concentration twice that of F1. Different letters indicate significant differences at P = 0.05.

Substrate	Total yield (g plant^-1^)	Marketable yield (g plant^-1^)	Fruit caliber (g plant^-1^)	Firmness	Sugar content	Leaf dry mass (g plant^-1^)
**CF**	8.46 (1.89) b	9.58 (2.38) b	11.99 (1.40) b	213.54 (25.08)	9.68 (0.58)	20.04 (4.27) b
**PS25**	7.18 (1.86) b	8.75 (2.43) b	10.70 (1.35) c	207.24 (13.63)	9.39 (0.65)	16.07 (3.94) c
**AB**	11.10 (2.78) a	13.20 (3.60) a	13.85 (1.30) a	201.18 (19.55)	9.09 (0.66)	27.49 (3.51) a
***P* value**	*** ***					
Substrate (S)	0.001	0.003	< .0001	0.336	0.101	< .0001
Fertilization (F)	0.408	0.373	0.092	0.263	0.836	0.122
S*F	0.571	0.473	0.254	0.489	0.777	0.170
* *	*** ***	*** ***				
***Contrasts***	*** ***	*** ***				
CF-PS25	0.147	0.415	0.001	0.448	0.28	< .0001
AB-PS25	0.011	0.008	< .0001	0.466	0.264	< .0001

#### Substrate salinity and nutrient immobilization

**Fig 5:**
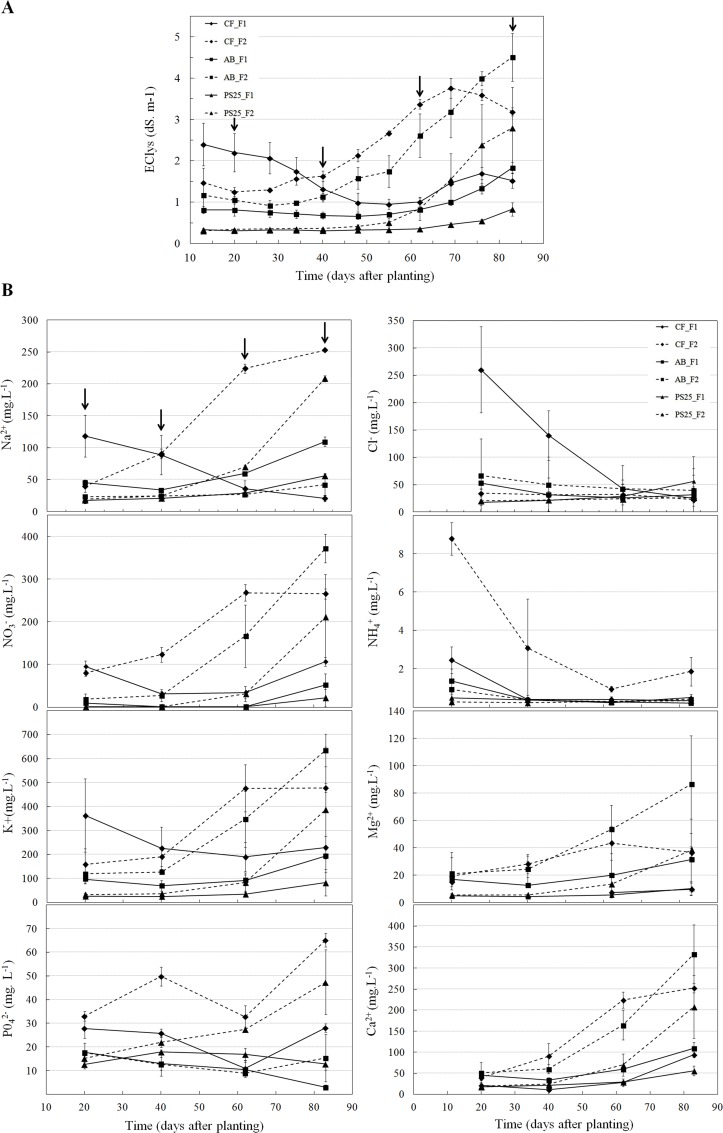
Variation in EC_lys_ **(A)** and plant-available nutrients **(B)** in the substrate water pore. The F1 condition (plain lines) and F2 condition (dotted lines) are represented for CF (green lines), AB (blue lines) and PS25 (red lines). Each point represents the mean (n = 3) with standard error (SE). The time points for which the nutrients were quantified are indicated by arrows.

Temporal evolution of EC_lys_ revealed a progressive accumulation of salts in the rhizosphere area during the course of the experiment (Fig 5A). For all substrates, EC_lys_ was significantly affected by fertilizer treatments from 8–9 weeks after planting without having a negative impact on fruit yield. EC_lys_ was higher for CF_F1 than CF_F2 at day 20, due to the high amount of sodium and chlorine in these coir fibers (Fig 5B). Our observations are in line with chemical properties of coir fibers which are known for their variable salinity and variable content of contaminants [21, 40, 54]. However, due to its low water retention properties, CF has been easily leached to eliminate the accumulated salts, thus decreasing EC_lys_ values to acceptable range in 34 days. As outlined above, this may have affected yield relative to the AB treatment though.

Cation and anion analyses revealed that the nutrient availability greatly differed among substrates (Table 6). Significantly lower amounts of K^+^, Mg^2+^, NO_3_^-^ and SO_4_^2-^ were observed with PS25 from day 20 (Table 6, Fig 5), showing early and high retentions of these elements. A significant substrate x fertilizer rate response was observed for Ca^2+^, Na^+^, NH_4_^+^, Cl^-^ and PO_4_^2-^. When the fertilization rate was increased, a significant increase in PO_4_^2-^ concentration was found with CF and PS25 but not with AB, suggesting a higher P retention rate with AB. A substantially higher level of PO_4_^2-^ was observed with CF compared to those with AB and PS25 at day 20 (Fig 5B), which was probably due to constitutive high levels of this ion in coconut coir substrates [54]. Increases in fertilizer rates resulted in increased amounts of Ca^2+^ and NH_4_^+^ concentrations with CF, whereas similar concentrations were observed amongst the two fertilizer treatments with AB and PS25. As a recent study highlighted the retention of Ca, Mg and P in coconut fiber- and woody fiber-based substrates [55], our results indicate (1) higher retentions of Mg^2+^ and Ca^2+^ with PS25 substrate compared to those with CF and AB (2), a higher PO_4_^2-^ retention rate with AB compared to those with PS25, while CF exhibited the lowest retention rate of this ion.

**Table 6:** Chemical properties and nutrients contents of substrate solution extracts were obtained by using a suction lysimeter during six treatments including those with CF, AB and PS25 substrates and two fertilizer programs (F1, F2). Means (n = 15) and SD are presented. Multiple comparisons were performed using the protected Fisher's *LSD* test, following one-way mixed model ANOVA. Significant levels of the *post hoc* test results are indicated by letters. Cations and anions were quantified after 20, 40, 62 and 83 d after planting in three independent blocks. The P-values obtained from the generalized linear mixed model (GLMM) used to fit the data are reported for the substrate (S), fertilization rate (F) and time (T) effects as well as their interactions. Significant interaction effects are indicated in bold.

Treatments	K^+^	Ca^2+^	Mg^2+^	Na^+^	Cl^-^	P0_4_^2-^	S0_4_^2-^	NH_4_^+^	NO_3_^-^
	(mg L^-1^)								
**CF**									
CF_F1	251.72 (107.15) a	38.80 (35.5) d	14.86 (10.57) cd	65.96 (53.64) a	117.15 (119.11) a	23.17 (8.59) bc	27.39 (19.4) bc	0.90 (1.09) b	66.95 (39.42) a
CF_F2	325.90 (167.38) a	151.74 (97.21) a	31.85 (11.3) ab	24.50 (11.13) bc	30.41 (17.49) bc	45.19 (15.00) a	58.59 (38.05) ab	3.66 (3.80) a	184.10 (90.43) a
**PS25**									
PS25_F1	41.78 (35.19) c	30.86 (18.69) bc	6.26 (3.51) e	18.53 (6.64) c	13.46 (5.45) d	15.03 (3.92) cd	18.09 (22.66) c	0.40 (0.24) b	6.11 (17.45) c
PS25_F2	134.28 (190.8) b	80.61 (98.82) ab	15.61 (17.46) d	26.87 (6.81) b	23.29 (4.84) c	28.03 (16.20) b	40.35 (40.60) b	0.28 (0.09) b	60.87 (118.2) b
**AB**									
AB_F1	112.51 (52.35) b	62.06 (33.43) d	20.19 (7.78) bc	22.39 (7.68) bc	35.63 (20.39) bc	11.05 (6.39) d	44.76 (37.87) ab	0.55 (0.68) b	15.78 (30.12) bc
AB_F2	306.95 (227.58) a	152.35 (133.35) cd	46.26 (32.78) a	29.18 (15.11) ab	49.95 (29.05) ab	13.62 (9.50) d	83.19 (77.14) a	0.50 (0.46) b	146.06 (161.23) a
***P value-LSD***	< .0001	< .0001	< .0001	0.0004	< .0001	< .0001	0.0006	< .0001	< .0001
***P value-GLMM***									
Substrate (S)	< .0001	0.002	< .0001	0.194	0.005	< .0001	0.001	0.002	< .0001
Time (T)	< .0001	< .0001	< .0001	0.701	0.037	0.041	< .0001	0.006	< .0001
Fertilization (F)	0.001	< .0001	< .0001	0.711	0.550	0.0001	0.0002	0.070	< .0001
S*F	0.082	**0.032**	0.337	**0.024**	**0.019**	**0.030**	0.764	**0.021**	0.074
S*T	**0.048**	0.924\	0.559	**0.007**	**0.049**	**0.0001**	0.140	0.145	0.241
F*T	**< .0001**	**0.009**	**0.012**	0.197	0.096	**0.010**	0.863	0.377	**0.0003**
***Contrasts***									
PS25_F1-PS25_F2	0.003	0.016	0.001	0.062	0.037	0.005	0.003	0.298	0.022
AB_F1-AB_F2	0.002	0.016	0.001	0.369	0.356	0.417	0.019	0.341	0.0002
CF-PS25	< .0001	0.021	< .0001	0.077	0.002	0.001	0.004	0.001	< .0001
CF-AB	0.004	0.067	0.006	0.303	0.644	< .0001	0.105	0.005	0.0001

While NH_4_^+^ was not detected with PS25 at both fertilization rates until day 62, early higher levels of this cation were observed with CF (Fig 5B). In comparison with CF_F1, consistently higher availabilities (about three times) were observed with CF_F2 and these could be the result of the increased fertilizer rate in addition to microbially-mediated Org-N mineralization followed by the release of the resulting NH_4_^+^ into substrate water pores. The amounts of NH_4_^+^ decreased between day 20 and day 40, probably due the nitrification of NH_4_^+^ to NO_3_^-^, a form which is more mobile and readily available to plants. NO_3_^-^ levels greatly differed between substrates and exhibited significant interactions between time and fertilization treatment (Table 6). With CF, plant-available NO_3_^-^ under F2 conditions increased with time, whereas it decreased under F1 conditions (Fig 5B). The significant decrease in these cations between day 20 and day 62 can be explained by NO_3_^-^ leaching below the root zone due to the intense drainage of the CF substrate, removing the excess salts. At both fertilizer rates, the NO_3_^-^ concentration at day 20 was lower with AB and PS25 compared to those with CF. Differences in NO_3_^-^ amounts between substrates can be explained by the existing N levels in the growing medium and/or N immobilization due to the activity of microorganisms. The C:N ratio is usually employed to indicate the maturity degree of compost and is a good indicator of nitrogen availability for the plants [56]. The C:N ratio was found to be low in peat substrates, intermediate in coconut coir substrates and high in bark material [46, 55, 57]. Woody materials generally exhibit acceptable C:N values for plant growth after composting and application of a initial fertilizers load [58]. Regarding peat-based substrates, Davis and collaborators [59] reported that a substrate composed of 80% peat and 20% sawdust had a C:N ratio of about 0.7, indicating that there is such a ready source of available nitrogen that they can be considered to be fertilizers. The lower N level observed with PS25 at the beginning of our experiment suggested an initial tie-up of N, probably resulting due to intense microbial activity. In contrast with PS25, lower existing N amounts in AB combined with a moderate microbial activity may have resulted in low N levels available to plants. Our results are consistent with previous studies that reported early/initial N immobilization in peat-lite and pine bark substrates [60]. Although previous studies have reported that N immobilization can occur in coconut fiber substrates [61], our results suggest a more important N immobilization with both AB and PS25 at the early stages of plant growth.

### Economical implications and environmental considerations

Plugs and bare-root plants are two planting methods commonly used for strawberry production. In spite of the many advantages associated with plug transplants, consistency in early fruit production is needed for plug plants to be economically cost effective, in comparison with bare-root plants [62, 63]. Previous research reported that the use of plugs or bare-root plants resulted in similar fruit yields in strawberry soilless culture [62]. In addition, effective water management and timely fertilizer application has the potential to reduce over-irrigation pumpage during bare-root plant establishment [64]. By taking these considerations into account, bare-root plant production using drip-irrigation systems appears a good alternative for strawberry growers because of planting flexibility, reduced costs and high plant quality. In our study, we aimed to use high tunnels as a means to provide the opportunity for local farmers to expand production windows of the day-neutral strawberry ‘Seascape’ in Quebec. Interestingly, similar growth and yields were obtained for plants grown with PS25 while saving 5.6–11% of water usage, compared to that with plants grown with AB and CF. The marketable yield reached in this experiment would enable local farmers to benefit and sell high quality strawberries for better prices.

Faced with increasing fertilizer prices and environmental problems due to the discharge of drained nutrient leachates to the environment, it is crucial for growers to be able to lower nutrient concentrations without affecting strawberry growth, fruit quality or yields [52]. In Quebec, we recommend the use of an irrigation EC of 1.0–1.5 dS m^-1^ by respecting element aspect ratio used in this study. We also showed that in spite of apparent N immobilization with AB and PS25- total N concentrations as low as 2.9–5.8 mmol L^-1^ during the vegetative period and 4.7–6.1 mmol L^-1^ during the fruit production period can be used for strawberry plants grown with PS25, AB and CF. These recommended concentrations fall in the range of previous values reported for strawberry plants grown in coconut coir and pine bark substrates in Brazil, where N levels were between 2.8 and 5.7 mmol L^-1^ [47].

Interestingly, our results showed that PS25, a locally-produced peat-based substrate, is a suitable substrate for strawberry soilless culture. However, an initial fertilizer application is required to limit the risk of nutrient tie-up and ensure the performance of this mixture. Because of its low cost and high availability in Canada, peat is a high value material which is expected to remain a major component of growing media over the next decades [22]. Even though this material is not readily renewable, the overall resources are important and major efforts are being made to reduce its carbon footprint by developing *Sphagnum* farming.

## Conclusions

The present study provides a useful guide for water and nutrient management for soilless strawberry production. Both PS25 and AB substrates appear suitable for soilless strawberry production. We have successfully established bare-root plants in a low cost peat-sawdust substrate and derived an appropriate irrigation strategy to obtain similar yields to plants grown in peat bark and coco fiber substrates in greenhouse and high-tunnel systems. The results obtained in Exp. 3 validate the concerns that were previously raised about the potential quality issues with coir-based substrates, and possible N tie up in sawdust based substrate. Taken together, our results imply that (1) salinity problems can cause management issues for coconut-based substrates and (2) sawdust- and bark-based materials can be used as substitutes to coconut coir for strawberry soilless culture in Canada, as long as an initial basic fertilization is applied to avoid the initial tied up observed in this study.

## Supporting Information

S1 Table Stock composition of the fertilizer solution used in the three experiments. (DOCX)

S2 Table Water use efficiency for fruit production and dry mass of the three substrates. (DOCX)

## Abbreviations

CF: coconut fiber
AB: aged bark
PS25: peat-sawdust
WP: water potential
WRC: water retention curves
AFP: air-filled porosity
WHC: water holding capacity
WBC: water buffering capacity
K_sat_: saturated hydraulic conductivity
BD: bulk density
EC: electrical conductivity
CEC: cation exchange capacity
Ka: dielectric constant value
h: matric potential
*θ*_*r*_: residual water content
*θ*_*s*_: water content at saturation
