# Supplementary material for: Sawdust and Bark-Based Substrates for Soilless Strawberry Production: Irrigation and Electrical Conductivity Management
Source: PLoS One. 2016 Apr 21;11(4):e0154104. doi: 10.1371/journal.pone.0154104 (PMC4839704; doi:10.1371/journal.pone.0154104)
Supplement: S1 Table — (DOCX) [file pone.0154104.s001.docx]

**Table S1.** Stock composition of the fertilizer solution used in the three experiments. The initial stock solution was prepared and diluted at EC 0.6 - 1.6 dS m-1 to fertilize the plants. N/A: not quantified; -: no fertilizer applied.
