# Supplementary material for: Sawdust and Bark-Based Substrates for Soilless Strawberry Production: Irrigation and Electrical Conductivity Management
Source: PLoS One. 2016 Apr 21;11(4):e0154104. doi: 10.1371/journal.pone.0154104 (PMC4839704; doi:10.1371/journal.pone.0154104)
Supplement: S2 Table — (DOCX) [file pone.0154104.s002.docx]

**Table S2.** Water use efficiency for fruit production and dry mass of the three substrates. Water use efficiency for plant dry matter (WUE_m_) was estimated as the ratio of the total dry mass to the water used during the whole plant growing period, while water use efficiency for fruit production (WUE_fp_) was calculated as the ratio of the cumulative total yield to water use.

| **Substrate** | **Water used**  **(L)** | **Total dry mass**  **(g)** | **WUE_m_ (g. L^-1^)** | **Cumulative total yield (g)** | **WUE_fp_**  **(g. L^-1^)** |
| --- | --- | --- | --- | --- | --- |
| CF | 70.72 | 1250.44 | 17.68 | 1057.13 | 14.95 |
| PS25 | 64.65 | 1304.5 | 20.18 | 1020.14 | 15.78 |
| AB | 72.58 | 1237.3 | 17.05 | 911.89 | 12.56 |
